# Precursors of Viral Proteases as Distinct Drug Targets

**DOI:** 10.3390/v13101981

**Published:** 2021-10-02

**Authors:** Taťána Majerová, Pavel Novotný

**Affiliations:** 1 Institute of Organic Chemistry and Biochemistry of the Czech Academy of Sciences, Flemingovo nám. 2, 166 10 Prague, Czech Republic; novotny@uochb.cas.cz; 2 Department of Physical and Macromolecular Chemistry, Faculty of Science, Charles University in Prague, 128 43 Prague, Czech Republic

**Keywords:** protease, autoprocessing, precursor, activation, Human Immunodeficiency Virus (HIV), Severe Acute Respiratory Syndrome Coronavirus 2 (SARS-CoV-2), herpesviruses, adenoviruses, flaviviruses

## Abstract

Viral proteases are indispensable for successful virion maturation, thus making them a prominent drug target. Their enzyme activity is tightly spatiotemporally regulated by expression in the precursor form with little or no activity, followed by activation via autoprocessing. These cleavage events are frequently triggered upon transportation to a specific compartment inside the host cell. Typically, precursor oligomerization or the presence of a co-factor is needed for activation. A detailed understanding of these mechanisms will allow ligands with non-canonical mechanisms of action to be designed, which would specifically modulate the initial irreversible steps of viral protease autoactivation. Binding sites exclusive to the precursor, including binding sites beyond the protease domain, can be exploited. Both inhibition and up-regulation of the proteolytic activity of viral proteases can be detrimental for the virus. All these possibilities are discussed using examples of medically relevant viruses including herpesviruses, adenoviruses, retroviruses, picornaviruses, caliciviruses, togaviruses, flaviviruses, and coronaviruses.

## 1. Precursors as Major Signalization and Orchestration Agents

Autoproteolytic processing of protease precursors releases the active enzyme and the adjacent sequence(s). These released peptides or proteins can have other specific functions. The earliest proteins discovered to be autoactivated are digestive enzymes such as pepsin, trypsin, or chymotrypsin [1,2,3,4,5,6,7]. The blood clotting cascade is an example of regulation via proteolytic autoactivation in the human body [8]. Inherent activity not only of the mature enzyme but also of the precursor form was first reviewed 50 years ago [1]. The first self-processing and self-activating enzymes were observed in 1966 [9].

Proteases often act as a trigger-point of biological processes and their activation via autoprocessing is irreversible. Not surprisingly, the self-cleavage of the precursor is tightly spatiotemporally regulated in order to occur in the correct location with optimal rates. In many cases, details of these processes remain unknown. Compartmentalization often plays a role in autoactivation, as exemplified by the lysosomal protease cathepsin D. The migration of procathepsin D to the lysosome leads to the release of the active protease from the precursor proenzyme due to the acidic pH of this compartment [10,11,12,13]. Autoprocessing also functions in signaling pathways when an N-terminal or C-terminal-signaling molecule is released from the precursor after self-cleavage. If a signaling molecule is present at both terminals, it can be involved in two independent regulatory pathways [14]. The propart (usually the sequence adjacent to the N-terminus of a protease) can have a intramolecular chaperone function and can guide proper enzyme folding [15].

### Viral Polyprotein Strategy

Viruses use various strategies for protein expression. One of these is to synthesize viral proteins, including one or more proteases, in the form of a polyprotein. Viral proteases are capable of autocatalytic release, along with cleavage of the viral polyprotein into separate functional proteins during the maturation process. Protein complex formation, timely differentiated steric accessibility of cleavage sites, and the interaction with host cell membrane organelles are all often involved in the regulation of proteolysis during viral maturation [16,17,18,19].

The polyprotein strategy is employed by positive RNA viruses, including retroviruses such as HIV (Human Immunodeficiency Virus) [17]; coronaviruses such as Severe Acute Respiratory Syndrome Coronavirus 2 (SARS-CoV-2) [20]; and by some DNA viruses, e.g., poxviruses (smallpox virus) [21] or herpesviruses [22]. Other DNA viruses, such as polyomaviruses [23,24], all negative RNA viruses (among them, influenza virus [25]), and double-stranded RNA viruses [26] use different strategies of protein expression and encode no protease.

Inhibition of a specific viral protease interrupts the viral life cycle by abolishing the production of viral proteins needed for the replication and spread of the virus. Active-site inhibitors of HIV and Hepatitis C Virus (HCV) proteases are in clinical use [17,27]. Compounds targeting viral proteases through a mechanism other than the inhibition of the active site could broaden the portfolio of antivirals to overcome the development of drug resistance and to solve problems regarding non-specificity, toxicity, or improper pharmacokinetic properties. The multifunctional nature of viral protease precursors offers several potential ways for antiviral therapeutic interventions. The strategies are discussed below.

## 2. DNA Viruses

### 2.1. Herpesviruses

Herpesviruses are enveloped DNA viruses causing diverse human diseases, such as cold sores or chicken pox. Some herpesviruses can establish persistent infections and are also important opportunistic pathogens. Several nucleoside derivatives targeting the viral replication machinery are in clinical use [28,29,30]. Efforts to develop inhibitors of the herpes protease-assemblinhave not yet yielded any clinical candidates.

**Figure 1:**
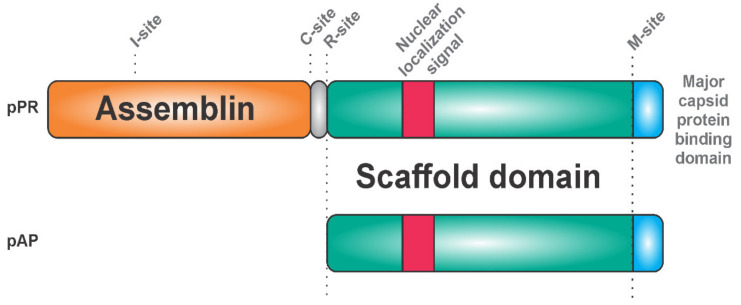
Herpesviruses: Two fusion proteins expressed from RNAs identical at the 3’-terminus, namely the protease precursor (pPR) embedding assemblin and the assembly protein precursor (pAP). The major capsid protein binding domain (blue) ensures the formation of a complex with the capsid protein. This complex is recruited into the cell nucleus via its nuclear localization sequence (red). Association of capsid proteins mediates the dimerization of assemblin, leading to its autoactivation. The scaffold protein is proteolytically processed at the M and R sites. The intra-assemblin processing sites I and C have regulatory roles.

Herpesviruses harbor a serine protease, which has a unique catalytic Ser-His-His triad and is active as a homodimer [31,32,33,34]. The protease is C-terminally fused with a scaffold protein, which has a major capsid protein-binding motif on its C-terminus [35,36] and a nuclear localization signal on its N-terminus (Figure 1).

The protease precursor pPR (expressed from the gene denoted as UL26) binds to the major capsid protein (this interaction probably inhibits its proteolytic activity) and results in the transportation of the whole complex into the cell nucleus, the site of virion assembly. In the nucleus, the scaffold domains of the protease precursor proteins self-associate into spherical procapsids, in which the protease domains come into its proximity [37]. Protease dimerization leads to conformational rearrangements, which result in a dramatic increase in proteolytic activity. Cleavage at two sites of the precursor (denoted as M and R) releases the scaffold protein. Detailed studies of the human cytomegalovirus (HCMV) also revealed two intra-assemblin cleavage sites [38]. Cleavage at the C site leads to assemblin inactivation, whereas processing at the I site generates non-covalently associated fragments of assemblin with retained activity [39]. Cleavage at the I site triggers structural rearrangements, which bury the C site and prevent its hydrolysis. Inhibiting hydrolysis at the C and I site by mutagenesis synergistically reduces virus infectivity by 90% [40]. It seems that the precise equilibrium between the active and inactive forms of assemblin is important for determining whether virus replication and spreading is successful.

Since the herpes virus protease is active in its dimeric form, disruption of inter-monomeric protein–protein interactions represents another option to design inhibitors. Peptides and alpha-helix mimetics were reported as inhibitors disrupting the dimerization interfaces between the two monomers [41,42,43]. Dimerization inhibitors might also act at the level of the pPR precursor. Compounds blocking protein–protein interactions of the pPR precursor in the scaffold domain could represent an interesting alternative for drug design. Even more speculative is the possibility of exploiting the scaffold domain for artificial premature activation of assemblin. Up-regulation of the proteolytic activity could be as detrimental for the virus as its down-regulation by classical inhibitors.

Experiments with purified enzymes showed that the protease can be overactivated. Specifically, artificial activation by cosmotropic agents, such as ammonium sulphate, increases the activity of assemblin by several orders of magnitude [44].

Another homologous herpesvirus protease with deubiquitinating activity was identified in a conserved area of the Herpes simplex virus-1 genome. This protease is embedded within the N-terminal, part of the large tegument protein UL36, and is active only after proteolytic release from its precursor [45,46,47]. It seems to be involved in the viral replication [48] and modulation of host cell antiviral responses [49,50], making it another viable drug target.

### 2.2. Adenoviruses

**Figure 2:**
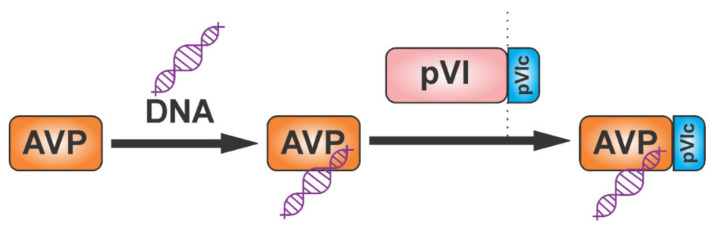
Adenoviruses are characterized by the activation of adenoviral protease (adenain); the activity of adenain (AVP) is stimulated by DNA and by the pVIc peptide released from the pre-pVI protein. Peptide pVIc forms a disulfide bond with adenain and, as a result, increases its proteolytic activity further.

Adenoviruses cause illnesses with symptoms similar to the common cold. They are non-enveloped DNA viruses that combine complex strategies of pre-mRNA splicing and protein processing to produce mature viral proteins [51,52,53,54]. The adenoviral protease adenain is translated from mRNA transcribed from the late L3 gene, together with two proteins important for assembly, namely pre-pVI and hexon. The adenoviral protease is activated by DNA and by the GVQSLKRRRCF peptide, which form a disulfide bond with adenain. This activating peptide is generated by proteolytic cleavage of the pre-pVI protein C-terminus [55,56] (Figure 2). The activation peptide binds far away from the active site. It triggers a bifurcated series of consecutive conformational changes involving 62 amino acids, leading to loop rearrangements and to the long-range communication of both the catalytic histidine and the phenylalanine of the activating peptide [57]. Adenain can cleave eight amino acids from the C-terminus of actin and the resulting peptide acts as an activator in a similar way to the viral activating GVQSLKRRRCF peptide [58].

When a synthetic version of the activating GVQSLKRRRCF peptide was added to adenovirus-infected cells in cell culture, the extent of virus production decreased [59]. This suggests that not only protease inhibition but also premature activation or dysregulation of the protease can be detrimental for the virus.

## 3. RNA Viruses

### 3.1. Retroviruses

Retroviruses are enveloped positive single-stranded RNA viruses that use retroviral reverse transcriptase to form a DNA intermediate and then integrase to incorporate the viral DNA into the host genome. Human immunodeficiency viruses (HIV-1 and HIV-2) and human T-lymphotropic viruses (HTLV-1 and HTLV-2) cause lifelong chronical infections in humans [60,61,62]. Fragments of various retroviruses found in the human genome document a wide range of past retroviral infections during history and evolution [63].

The epidemic of HIV in the 1980s led to an intensive effort in the field of antiviral drug development. The first antivirals in clinical use were nucleoside derivatives interfering with the synthesis of proviral DNA, the first being specifically azidothymidine (zidovudine) in 1987 [64,65,66]. The first antivirals with a completely novel mechanism of inhibition were inhibitors of the HIV protease. The earliest of them, saquinavir, was approved by the U.S. Food and Drug Administration (FDA) in 1995 [67,68,69]. The simultaneous targeting of two different steps of viral replication (the synthesis of proviral DNA and the cleavage of viral polyproteins into functional proteins) started a new era in the treatment of AIDS/HIV: the highly active antiretroviral therapy (HAART). Compounds blocking other steps of the viral life cycle followed [70]. Cabotegravir [71,72], a strand transfer HIV integrase inhibitor [73] approved in 2021, is the most recent one among them. HAART changed HIV infection from a fatal to a chronic manageable disease, although the virus remains integrated in host cells. To minimize the risk of evolution of drug-resistant variants [74,75,76,77,78,79,80,81,82], sophisticated drug combinations and the precise dosage are important [83,84,85]. The treatment in individual patients must sometimes be changed not only due to the development of drug resistance but also due to unwanted side effects appearing after long-term therapy [86]. Thus, new compounds with different chemical structures, diverse binding modes, or with novel unique mechanisms of action are continuously desired.

**Figure 3:**
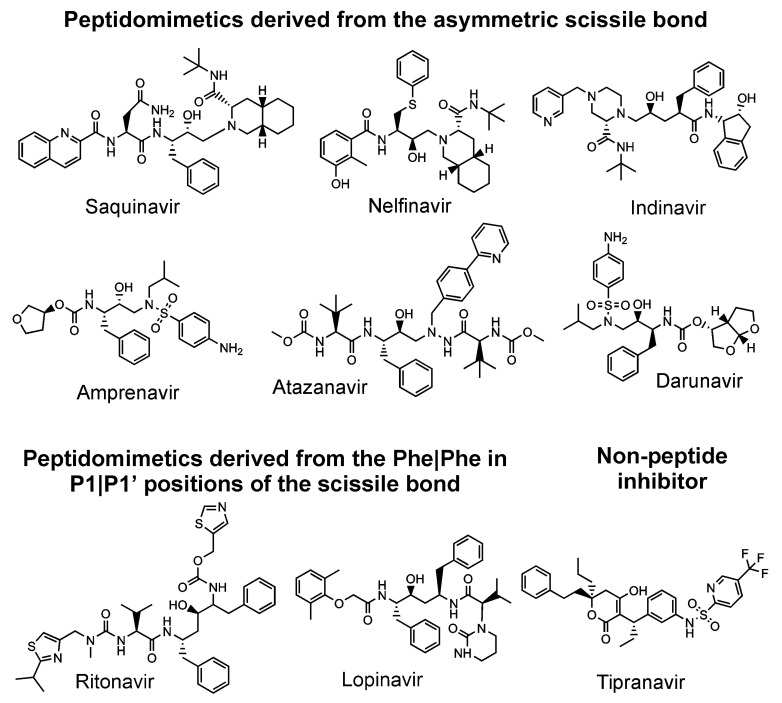
FDA-approved inhibitors of the HIV protease.

All nine HIV protease inhibitors in clinical use (saquinavir, indinavir, ritonavir, nelfinavir, amprenavir, lopinavir, tipranavir, atazanavir, and darunavir) target the active site [17,79] of the HIV protease, although it has been suggested that darunavir [87] and tipranavir [88] may also block dimerization via a secondary binding site (Figure 3). It has been shown that all these inhibitors have an affinity for the precursor, which is of several orders of magnitude lower than the affinity for its mature form [89,90,91,92]. Thus, an inhibitor targeting the precursor would block the first rate-limiting step of the protease cleavage and maturation cascade [93]. Such a compound could have a higher barrier to drug-resistance development due to its expected ability to conformationally bind loose structures such as precursors and drug-resistant mutants [94]. Darunavir is the most potent inhibitor of the precursor forms of the HIV protease [89,92,95,96]. At the same time, this compound has a very high barrier against drug-resistance development [97].

**Figure 4:**
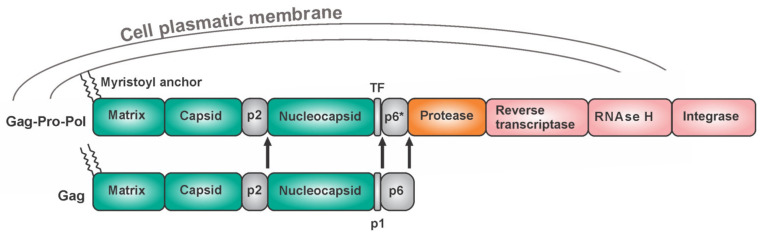
Retroviruses: Gag and Gag-Pol polyprotein of HIV-1 with its N-terminal myristoyl membrane anchor. The ratio of the Gag and Gag-Pol molecules is precisely regulated by the frequency of frameshift events. The arrows indicate the initial cis-cleavage sites.

The HIV protease is an obligatory homodimer. Attempts to design dimerization inhibitors have not revealed any clinical candidates [98,99,100,101,102,103]. The HIV protease belongs to the family of aspartic proteases. Each monomer provides one catalytic triad, namely Asp-Thr-Gly, which forms the active site through a network of hydrogen bonds, an arrangement which is referred to as the “fireman grip” [104,105]. The HIV protease is expressed in vivo as part of the Gag-Pol polyprotein (Figure 4). The Gag region harbors structural proteins (matrix, capsid, and nucleocapsid), while the Pol region bears viral enzymes. Gag is expressed separately approximately 95% of the time. After a-1 ribosomal frameshift, Gag is produced in fusion with Pol. This occurs in 5% of the translating events. Keeping the optimal Gag to Gag-Pol ratio is critical for the successful production of viral progeny [106]. One monomer of the HIV protease is embedded in each Gag-Pol macromolecule. To cleave itself out of the precursor, two Gag-Pol moieties must form at least a transient dimeric structure to form the active site. The initial cleavage does not release the mature protease but instead, the first hydrolysis reactions occur intramolecularly (in cis) between the p2 peptide and the nucleocapsid, and between the transframe peptide (TFP) and the p6* peptide, followed by cis N-terminal removal of HIV-1 PR from the precursor (Figure 4). The subsequent steps of protease maturation are intermolecular (trans cleavage) [107,108,109]. The HIV protease occurs in several forms—as the precursor as partially processed polyproteins and as the mature form. In general, partially processed and unprocessed peptides of amino acid sequences found adjacent to the protease region might influence the protease domain, thus affecting its dimerization ability [109,110], substrate specificity, accessibility of cleavage sites, structural features, and stability. Among other retroviruses, as many as three C-terminally truncated mature forms of Mason–Pfizer monkey virus (M-PMV) protease were reported to exist and these have different levels of activity and stability [111,112,113,114,115].

Protease-inhibitor resistant variants harboring mutations not in the protease but in the Gag region [80,116] have been reported and protease mutants change the susceptibility to bevirimat, which is a maturation inhibitor that binds the Gag polyprotein [81]. Gag and Gag-Pol most probably contain intrinsically disordered regions that enable their functional plasticity [117]. The precursor forms may include transient ligand binding sites, which would disappear after maturation and could be targeted by novel drugs.

**Figure 5:**
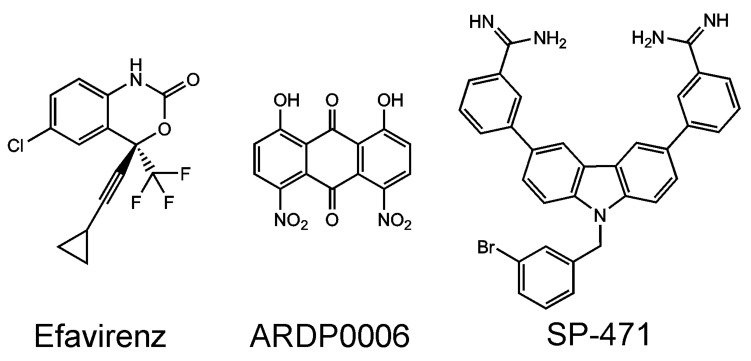
Compounds binding precursors of viral proteases: efavirenz binds to the Gag-Pol precursor, leading to the premature activation of the HIV protease, whereas ARDP0006 and the carbazole derivative SP-471 block intrahelicase cis-cleavage in Dengue NS3.

Some non-nucleoside HIV reverse transcriptase inhibitors induce premature HIV protease activation [118]. Namely, efavirenz (Figure 5)—a reverse transcriptase allosteric inhibitor—has an adverse effect on the production of viral particles. It works by bringing the reverse transcriptase domains of two Gag-Pol molecules into close proximity, which is followed by the dimerization of the two protease domains and ultimately leads to premature autoprocessing of HIV protease. Premature cleavage of the Gag-Pol molecules impairs the production of viral particles [119]. This effect was observed in the micromolar concentration range in tissue culture. Such a high concentration of efavirenz is probably well above a safe therapeutic concentration and, moreover, is unlikely to be reachable in vivo. Thus, this finding is not directly applicable in therapy. It represents a valuable proof-of-concept, encouraging the search for more efficient compounds triggering the premature activation of the HIV protease as a completely novel class of drugs. The HIV protease is cytotoxic [92,120,121,122,123] and its premature activation in the cytoplasm of host cells could lead to the elimination of infected cells with HIV integrated in their genome [124,125], thus acting as a causative cure. It is also known that allosteric integrase inhibitors, promoting multimerization of HIV integrase, impair not only integration but also particle core maturation as well as reverse transcription during the subsequent round of virus infection [126]. The influence of allosteric integrase inhibitors on the HIV protease and its processing was not studied to date.

Highly active HIV protease variants seem to be evolutionarily unfavorable. Viral constructs bearing two copies of the HIV PR monomers genetically tethered in one Gag-Pol polyprotein do not produce infectious viral progeny since the premature processing of the viral polyproteins prevents viral particle formation and infectivity. Premature processing also leads to increased cell toxicity [127]. Similarly, the placement of leucine zippers at the C-terminus of HIV PR in viral constructs to force the formation of enzymatically active PR dimers significantly reduced the production of virus-like particles. The production of virus-like particles was restored to wild-type levels by the addition of HIV PR inhibitors [128]. Deletion of the transframe region—a sequence adjacent to the N-terminus of the HIV protease, preventing premature HIV PR autoprocessing—led to the production of virions with improperly processed polyproteins and greatly reduced viral infectivity [129]. Impaired capsid assembly after premature viral protease activation was also reported for Mouse Mammary Tumor Virus [130].

Gag [131,132] and Gag-Pol polyproteins are recruited to the cell membrane, which is the site of viral assembly [133]. In HIV, the N-terminal myristoyl moiety of Gag anchors viral proteins to the cell membrane. Inhibition of myristoylation [134] or blocking of the interaction of viral polyproteins with the host cell membrane could prevent proper virion assembly [135]. It could also mediate in-cell protease activation, supporting the elimination of virus-infected cells through the cytotoxicity of the HIV protease.

### 3.2. Picornaviruses

Picornaviruses are the causative agents of diseases such as polio, rhinitis, or hepatitis A [136]. They belong to the family of positive-strand RNA non-enveloped viruses and possess a chymotrypsin-like protease with a catalytic cysteine in its active site. This protease is denoted as the 3C proteinase or picornain [137]. It co-translationally and post-translationally cleaves the nascent viral polyprotein. The rate and order of hydrolysis events are precisely tuned and are linked to viral replication. Additionally, not only do the fully processed mature viral proteins have specific functions but the partially cleaved intermediates also play a distinct role, which is indispensable in the viral life cycle.

Picornaviral polyprotein P3 harbors four proteins: 3A [138] (hijacks host factors), 3B (important for priming of RNA synthesis), 3C (protease), and 3D (RNA polymerase). The 3C protease possesses a protease domain with an RNA-binding domain on its surface. The 3CD precursor (fusion of the protease and RNA polymerase) has proteolytic activity, which is several orders of magnitude higher than that of the mature 3C protease when measuring the rate at which structural proteins and 3CD are processed. The other non-structural proteins are cleaved by 3C and 3CD with a comparable efficiency [139]. Although the X-ray structure of mature 3C, 3D, and the precursor 3CD do not show distinct differences between the processed and unprocessed forms [140], changes in the conformational dynamics between free 3C and 3D proteins compared with 3CD have been observed [141]. Synthesis of viral RNA is primed by a covalent link between viral RNA and the 3B protein (also denoted as VPg). P3 molecules recruit other P3 molecules to the replication complex. VPg and the 3D RNA polymerase, which is active only in its active form, are then released by proteolytic cleavage, enabling the synthesis of viral RNA [142]. Interaction of the 3C protease with RNA and with the VPg protein resulted in conformational changes of the RNA-binding site and the active site, indicating long-range allosteric communication between these two sites [143]. Studies with inhibitory antibodies revealed two potential allosteric binding sites, which are conserved and could be used for further drug design [144]. Allosteric compounds and protein–protein/RNA interaction disruptors can represent interesting strategies for picornain-targeting.

The initial cleavage of the full-length viral polyprotein can be catalyzed in cis by a separate protease 2A. This strategy is employed by some picornaviruses of the Rhinovirus and Enterovirus genera. The 2A protease is also a chymotrypsin-like cysteine proteinase and plays a role in the evasion of the host immune response [145]. Viruses belonging to the Aphtovirus and Cardiovirus genera perform the first cleavage using a non-enzymatic mechanism [146].

### 3.3. Caliciviruses

Caliciviruses cause gastroenteritis in humans [147] and share similar features with picornaviruses. Their viral polyprotein harbors the NF6 protease, which generates partially processed fragments with specific and temporally defined roles in the life cycle, along with mature proteins [148,149,150]. This strategy of using multipurpose proteins is useful for saving genomic space. From the point of view of drug design, targeting one protein can disrupt several different steps in the life cycle.

### 3.4. Togaviruses

Togaviruses have two genera: Rubivirus (harboring the causative agent of rubella) and Alphavirus. Alphaviruses are positive-enveloped RNA viruses that cause a variety of neglected tropical diseases, including chikungunya [151]. Their viral non-structural proteins are expressed as polyprotein NSp1-NSp2-NSp3 or NSp1-NSp2-NSp3-NSp4 when the stop codon between NSp3 and NSp4 is suppressed [152]. The protease is located in the C-terminus of NSp2 together with an N-terminal helicase. The first cleavage event during protease maturation occurs in cis or in trans between NSp3 (macrodomain) and NSp4 (RNA polymerase), and is followed by the release of NSp1 (mRNA capping enzyme [153]), solely cleaved in cis [154]. In addition to having RNA-modifying activity, NSp1 functions to anchor the replication complex to the host cell membrane and helps the formation of membrane vesicles, which protects the viral replication process from being blocked by the host cell defense machinery. NSp1 monomers associate into a ring structure formed by dodecamers [155]. It is possible that oligomeric structures are also formed by the unprocessed or partially processed precursor polyproteins and could thus drive both the formation of virus-induced membrane microcompartments and the synthesis of viral RNA [155,156]. The complex of NSp1, NSp4, and the fusion protein NSp2-NSp3 synthesizes a negative RNA intermediate [157,158]. The last proteolytic cleavage occurs between NSp2 and NSp3, releasing fully mature enzymes and probably changing the conformation of the RNA-binding surface. This structural change switches the replication complex to synthesize the positive RNA strand. The cleavage between NSp2-NSp3 is driven by a steric change, making the scissile bond accessible. Mutations in NSp3 at the sites of contact with NSp2 lead to decreased production of viral RNA [159]. Targeting of the key protein–protein interactions by small molecule compounds (specifically between NSp2 and NSp3) could disrupt viral replication. Artificial mutations in cleavage sites identified variants that were cleaved more efficiently than wild-type variants [160]. An overactive NSp2 protease variant has been reported [158]. Accelerated processing of viral polyproteins, either due to mutations in the cleavage sites or due to increased enzyme activity, led to a decrease in the production of infectious virions [158,160,161]. This suggests that the optimal cleavage rate does not equate to the maximal cleavage rate and that the natural sequence of events represents a compromise between the cleavage rate and other factors required for successful viral production.

### 3.5. Flaviviruses

Flaviruses are enveloped positive RNA viruses. Dengue, Zika, West Nile, yellow fever, and tick-borne encephalitis viruses are all members of this family. A more distantly related member of this family is the hepatitis C virus (HCV).

The genetic information of flaviviruses is translated into a single polyprotein harboring three structural (capsid C, membrane precursor prM, and envelope E) and seven non-structural proteins with enzymatic or accessory functions [162]. The viral polyprotein is threaded back and forth through the membrane of the endoplasmic reticulum. The co-translational and post-translational cleavage of the polyprotein is performed by host proteases on the luminal side (at the prM/E, E/NS1, NS1/NS2A, and NS4A/NS4B junctions) [163,164] and by the viral NS2B/NS3 protease on the cytosolic side (at the C/prM, NS2A/NS2B, NS2B/NS3, NS3/NS4A, and NS4B/NS5 junctions) [165,166]. For capsid formation to occur, exact temporally coordinated orchestration of the polyprotein cleavage by the NS2B-NS3 viral protease and by host proteases is necessary [167,168]. Immature virions are formed in the Golgi apparatus, prM is cleaved by furin, and envelope glycoproteins undergo a rearrangement to complete viral maturation [169,170,171].

**Figure 6:**
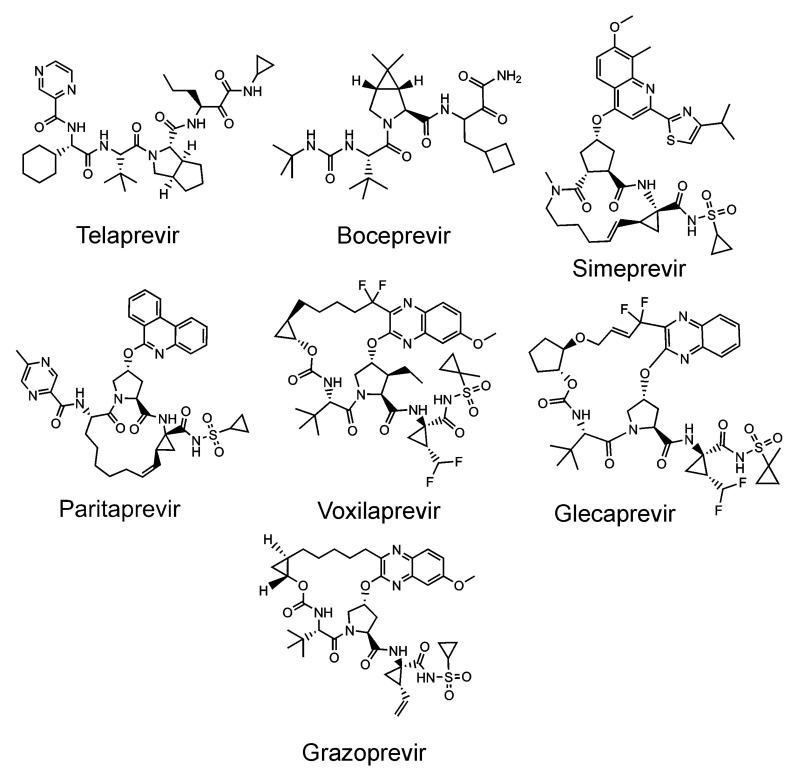
FDA-approved inhibitors of the HCV protease.

The flaviviral protease consists of the NS3 catalytic domain and the NS2B peptide cofactor for viruses of the Flavivirus genus [165], or the NS4A cofactor in the case of HCV (of the Hepacivirus genus) [172]. The introduction of inhibitors targeting the HCV protease (Figure 6) was a breakthrough in hepatitis C therapy [27,173]. These inhibitors are peptidomimetics targeted against the active site. As with other antivirals, the development of drug resistance represents a potential threat. Inhibitors of no other flaviviral proteases have been introduced into the clinic to date [174,175].

**Figure 7:**
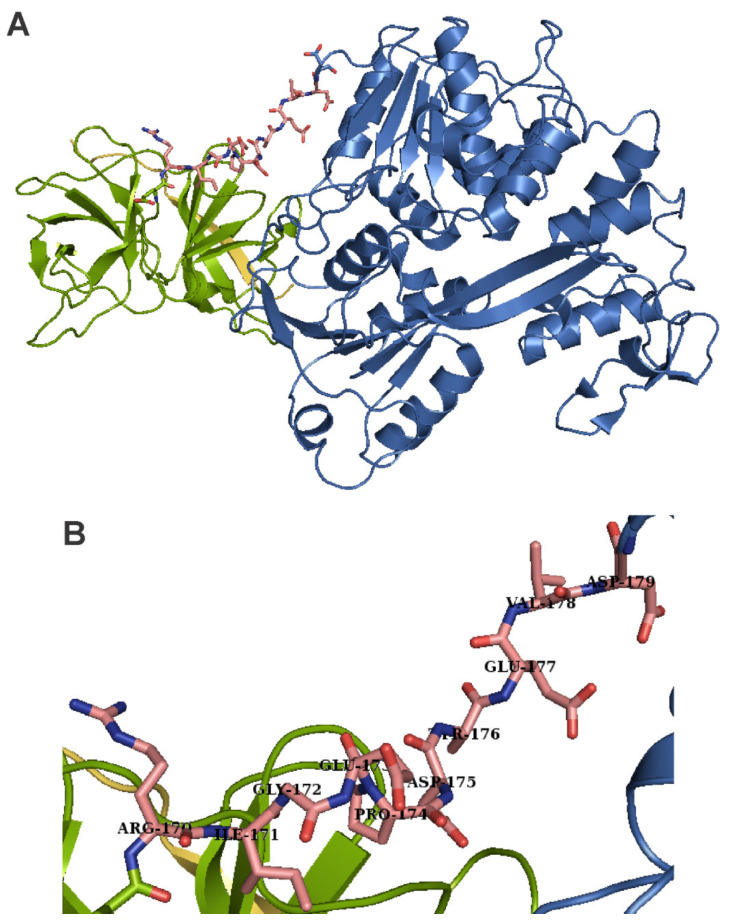
Overall structure of the Dengue NS2B-NS3 heterodimer, which harbors the protease needed for releasing viral proteins from the precursor (**A**). Close-up of the linker connecting the protease and helicase domains of NS3 (**B**). The protease domain is shown in green, the helicase domain is colored in blue, and the activating peptide of NS2B is yellow. The ten amino acids linker, connecting the two enzymatically independent domains of NS3, is shown in pink (carbon backbone). Within the linker, oxygen and nitrogen atoms are shown in red and blue, respectively. The PDB accession number of the structure shown is 2VBC [193].

The flaviviral chaperon-like activating peptide NS2B protrudes from the ER membrane, helping to form a chymotrypsin-like structure within the NS3 protease. [176]. A residual activity of solitary NS3 was reported [177]. The catalytic Ser-His-Asp triad is located in the cleft between two β-barrels [178]. Cleavage between the NS2B and NS3 proteins may not be necessary for proper activity, as during in vitro studies, the NS2B-NS3 protease was active as a fusion protein connected with a polyglycine linker between the NS2B and NS3 domains [179,180]. Compared to the NS2B-NS3 fusion protease, the non-covalently associated heterodimer formed by the NS2B and NS3 subunits might be oriented differently towards the cell membrane or may have some subtle differences in its substrate preference, which may be important for orchestrated polyprotein precursor cleavage [181]. The NS3 protein of flaviviruses contains both the protease and the helicase domains. The helicase domain has NTPase and nucleic acid unwinding activity. Despite the helicase domain having no effect on protease activity, the presence of the protease domain improves RNA unwinding activity in Kunjin virus NS3 [182] as well as in Dengue virus NS3 [183]. ATP hydrolase and RNA triphosphatase activity (catalyzed also by the helicase domain) were influenced by neither the presence nor the absence of the protease domain in Kunjin virus NS3 [182]. The ATPase active site of Dengue virus has lower affinity for ATP when the protease domain is present [184]. This can be explained using the structure of the full-length flaviviral NS3 (Figure 7). The connection between the protease and helicase domains is in contact with the NTPase domain active site, which is blocked by the linker between the protease and helicase. This might result in the reduced accessibility of the active site for ATP [180].

Since the active site of the flaviviral protease is flat and would need to be inhibited with charged ligands, whose transportation across the cell membrane is likely to be problematic, alternative antiviral strategies could be used, such as allosteric inhibitors [185] or disruptors of protein–protein interactions.

Apart from the canonical interprotein cleavage sites, another sequence of the intramolecular cleavage event is needed for the temporal orchestration of the viral life cycle [186,187]. It has been shown that at least three cleavages occur exclusively in cis (i.e., intramolecularly) at sites flanking the NS3 and at an internal cleavage site within the NS3 helicase. When the intrahelicase cleavage site was blocked by mutagenesis, the accumulation of unprocessed precursors inhibited the production of infectious viral particles in trans (i.e., intermolecularly). The inhibitory effect was trans-dominant when cells were co-infected by wild-type mutants and by an autoprocessing defective mutant [188]. ARDP0006 (1,8-dihydroxy-4,5-dinitroanthraquinone), an inhibitor of the Dengue virus-2 (DENV-2) protease, has been reported to inhibit virus replication in tissue culture in the low micromolar range [189]. Using in vitro enzyme kinetic measurements with purified enzymes, ARDP0006 was several orders of magnitude weaker than in the tissue culture [190]. Experiments have shown that ARDP0006 has a much higher affinity for the precursor form of the DENV-2 than for its mature form and that this compound preferentially blocks intrahelicase cis-cleavage. The accumulation of unprocessed precursors inhibited the production of infectious viral particles in trans (intermolecularly). The accumulation of precursors also blocked the production of mature virions in trans. Thus, the inhibitory effect of ARDP0006 can persist even if a cell harbors a mix of susceptible and ARDP0006-resistant viral variants [188].

Multimodal inhibitors with the carbazole scaffold, which block cis as well as trans cleavage events by the Dengue protease, have been reported [191] (Figure 5). Targeting of the protease precursor, exclusively autoprocessed in cis, represents an interesting strategy for the counter-development of drug resistance.

Another approach used the Dengue protease as a suicide initiator to activate a prodrug, resulting in the release of the cytotoxic combretastatin. This led to selective killing of virus-infected host cells [192]. Suicide protease substrates represent another alternative therapeutic strategy useful for the elimination of virus-infected cells.

### 3.6. Coronaviruses

**Figure 8:**
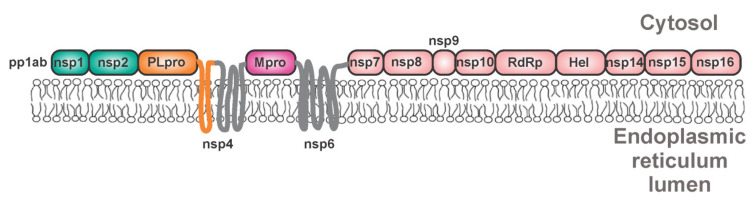
Polyprotein precursor pp1ab of SARS-CoV-2. Precursors of coronaviral proteases are anchored to the membrane of the endoplasmic reticulum by adjacent transmembrane domains of neighboring proteins.

Coronaviruses are detrimental human and animal pathogens [194]. These single-stranded positive RNA-enveloped viruses cause respiratory, enteric, neurological, and hepatic diseases. Including novel SARS-CoV-2, seven human pathogenic coronaviruses were identified [195]. Coronaviruses possess four major structural proteins: spike (S), envelope (E), membrane (M), and nucleocapsid (N), all translated from subgenomic RNAs [196]. The viral RNA is about 29.8 kb long and is translated into two polyproteins, namely pp1a and pp1ab [197] (Figure 8). These polyproteins are cleaved by two or three viral cysteine proteases, by the chymotrypsin-like main protease (Mpro, 3CLpro, and nsp5), and by one or two papain-like proteases. SARS-CoV-2 possesses one of each type of these proteases. The papain-like protease (PLpro and nsp3) releases the first three proteins: nsp1, nsp2, and nsp3 [198]. Mpro releases the remaining 13 non-structural proteins (including itself) [199]. The viral non-structural proteins are produced as polyprotein 1a (bearing proteins nsp1–nsp5) or polyprotein 1ab, which result from a frameshift (Figure 8) [200].

Mpro is activated via dimerization and its monomeric form is nearly inactive [201]. Oligomerization can further increase activity as seen during in vitro studies with a highly active octamer of Mpro [202]. Despite its dimerization, molecular dynamics studies suggest that only one protomer is active within the Mpro dimer [203]. Both active sites of the dimer are occupied by inhibitors in X-ray structures. Symmetrical as well as asymmetrical 3D structures of dimers were reported for the SARS-CoV-2 Mpro complex with an inhibitor. In the asymmetric dimer, one monomer exhibited inactive conformation of the active site [204,205]. The assumption of asymmetry of the SARS-CoV-2 Mpro dimer was supported by molecular dynamics simulations [206] and implies allosteric cooperativity between subunits [205,207].

The release of the mature protease from the precursor is initiated at the N-terminus of Mpro, presumably in cis in an intra-dimer and inter-protomer manner (i.e., two unprocessed monomers form a dimer and one monomer cleaves the second monomer), or in trans, later followed by C-terminal cleavage occurring in trans [208,209,210,211,212,213]. Structural “snap-shots” of cleavage and dimerization events were obtained during crystallization trials [211,213]. Amino acids adjacent to the N-terminus of Mpro influence the conformation of the active site by preventing dimerization, thus diminishing its activity. Potentially druggable ligand binding sites were identified in the precursor, which do not occur in the mature enzyme [211]. Studies with artificial peptide substrates based on SARS-CoV natural cleavage sites showed that the peptides corresponding to the cleavage sites adjacent to Mpro are hydrolyzed much more efficiently than peptides derived from the remaining cleavage sites [214]. The same conclusion was reached when calculating catalytic efficiency from data obtained from in vitro assays with N and C-terminal mutants [209]. These mutants, designed as uncleavable pro-forms of the wild-type enzyme with mutated N and/or C-autoprocessing sites, cleaved a proteolytically inactive precursor with authentic N and C-terminal flanking regions. The mature protease was inhibited by the precursor form with an active site mutation due to cross-dimerization of the mature and mutated pro-form of the protease [209].

For SARS-CoV, it has been found that dimerization-defective mutants (Arg4Glu, Glu290Arg, and Arg298Glu) can be autoprocessed on the N-terminus in cis but are not active in trans in vitro. An active-site mutant of the dimerization-defective variant was cleaved by the mature dimerization-defective mutant, indicating that N-terminal autoprocessing requires only a transient dimerization without a fixed conformation of the mature active site [210]. In particular, mutation of Arg298 results in a monomeric form of Mpro, leading to an inactive conformation and eventually to irreversible collapse of the substrate pocket [215]. The key residue connecting the substrate binding and dimerization events is Glu166 [216]. This residue is responsible for recognizing Gln in the P1 position of substrates and for the interaction with the S1 pocket of the heterologous protomer [208]. Dimerization-defective mutants can presumably be stabilized after substrate binding. When Glu166 is mutated, this mode of dimerization vanishes [216]. Dimer stabilization by substrates was reported also for other viral proteases [217,218].

Hyperactive variants of SARS Mpro have been reported. The mutations of Ser284-Thr285-Ile286 and Phe291 to Ala lead to increased enzyme activity without an apparent effect on its structure and dimerization. It was concluded that these critical residues form a nano-channel acting as an allosteric regulator [219,220,221].

**Figure 9:**
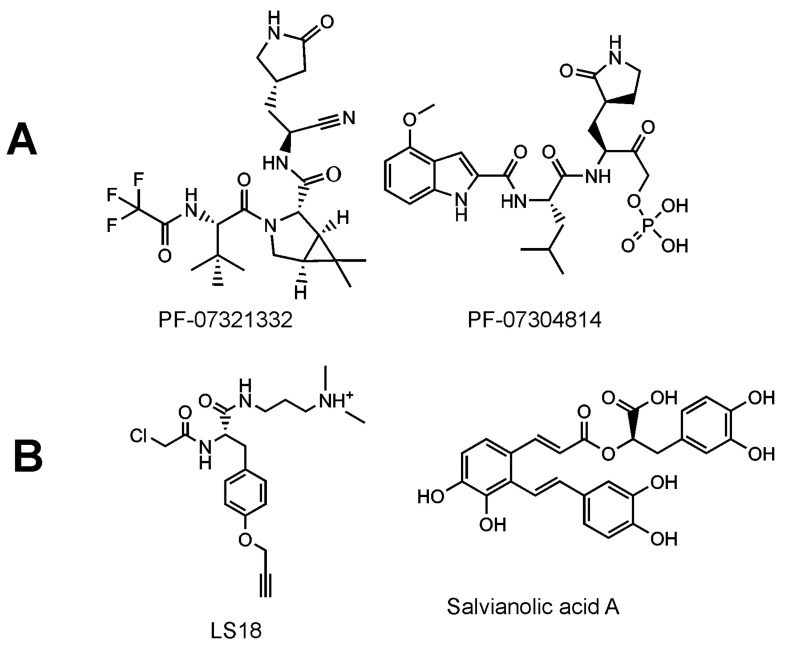
Inhibitors of SARS-CoV-2 Mpro in clinical trials (**A**) and inhibitors of the Mpro precursor (**B**).

Inhibitors of SARS-CoV-2 targeting the Mpro improved outcomes in a mouse model of coronaviral infection and increased survival of mice [222]. Potent inhibitors of SARS-CoV-2 have been reported, including GC376, a bisulphite adduct of a peptide aldehyde (preclinical studies have been initiated by the Anivive Lifesciences Company, Long Beach, CA, USA) [223,224], and a hydroxymethylketone derivative, specifically PF-07304814, which is an oral drug candidate of Pfizer [225] (Figure 9). The clinical candidates are prodrugs. Upon activation, the inhibitors are targeted against the Mpro active site, forming a covalent bond between the catalytic nucleophilic cysteinevia an electrophilic warhead. The key structural feature seems to be the γ-lactam glutamine surrogate at the P1 position. Recently, another inhibitor of Mpro, denoted as PBI-0451, has entered into phase 1 clinical trials (ClinicalTrials.gov registration number: NCT05011812).

Various inhibitors and strategies are under investigation. The attention was first focused on the potential repurposing of approved medicines [226,227,228] and masitinib, a tyrosine kinase inhibitor, has been identified among them [229]. Another strategy is represented by de novo designs of active-site [230,231] and allosteric ligands [232]. Natural products, such as flavonoids and their derivatives [233,234] or terpenes, were shown to be inhibitors of Mpro. For example, in addition to its inhibitory activity at submicromolar concentrations, the terpene eugenol promoted the oligomerization of Mpro in vitro [235].

The evaluation of the effect of Mpro inhibitors on the precursor forms of the protease has not yet received sufficient attention. Inhibition of the pre-processed Mpro has been attempted in vitro using a non-cleavable N-terminal Strep-tagged variant of Mpro as a model precursor. Click chemistry was used to fluorescently label the ligands covalently bound to the active site. Next followed SDS (sodium dodecyl sulfate) electrophoresis to separate the inhibitor-bound model precursor of Mpro and the fluorescence intensity of the band corresponding to the model precursor of Mpro was evaluated. A compound derived from tyrosine, harboring a chloroacetamide warhead, was identified as a precursor inhibitor. This compound was used as a probe in a competitive screen and salvianolic acid A was evaluated as a most-potent compound inhibiting in the low-micromolar range (Figure 9) [236]. The active site of SARS-CoV-2 Mpro seems to be structurally malleable [236,237,238], a feature which could be more pronounced in the case of the precursor forms. Targeting the unprocessed protease would block the maturation process during the first irreversible step. Even if such an inhibitor was removed, this might not lead to a recovery of viral maturation, as indicated by experiments with the HIV protease [239]. Dimerization inhibitors and allosteric ligands binding to the precursor form could also be interesting first-in-class drugs targeting SARS-CoV-2 Mpro.

In beta coronaviruses, a large 213 kDa nsp3 harbors a 36 kDa papain-like protease. This protease self-releases nsp3 out of the viral polyprotein and cleaves host protein substrates [240]. For self-cleavage at the nsp3/4 boundary, membrane association of nsp3 might be required [241]. The proteases probably self-cleave later in the coronaviral life cycle because the intermediates nsp2-nsp3 and nsp4-nsp11 were identified in SARS-CoV and Mouse hepatitis virus-infected cells [241,242]. Intermediates between the precursor and the mature protein are also predicted to be participants in the viral replication cycle [241,243].

The coronaviral papain-like protease (PLpro) possesses specific structural features, namely the palm, thumb, finger (containing a zinc ion), and a ubiquitin-like domain [244]. The finger and palm domains are important for interactions with ubiquitin, with this interaction being the first step of deubiquitylation activity [245]. The papain-like protease also releases the ISG15 protein, thus modulating the innate host immune response [246]. PLpro can also stabilize an E3 ubiquitin ligase and help ubiquitinate p53 [247]. Predicted inhibitors of PLpro, such as disulfiram [248,249] (ClinicalTrials.gov registration number: NCT04485130) and isotretinoin (ClinicalTrials.gov registration number: NCT04361422), are both in Phase 2 clinical trials due to their potential for repurposing.

A possible overlap of coronaviral papain-like proteases with substrate specificities of host deubiquitylating enzymes makes it difficult to design active-site inhibitors. Highly specific active-site inhibitors are therefore desired [250]. Allosteric modulators and other compounds with alternative mechanisms of action could be more suitable to avoid off-target binding. Yeast-surface display nanobodies (a single-domain antibody) were developed as potential inhibitors of PLpro [251]. This strategy represents an interesting alternative to classical inhibitors.

## 4. Concluding Remarks

Inhibitors of HIV and HCV proteases are important components of current antiviral therapies. These drugs primarily bind to the enzymes’ active sites. Proteases from other viruses can be exploited for therapeutic intervention as well.

Targeting viral proteases in their precursor form brings several possibilities for the development of compounds with unique mechanisms of action. Inhibitors binding to the precursor form would block the first rate-limiting step of the “domino” cascade, leading to viral maturation. Such compounds would bind to the active site of a precursor (which can differ in terms of the substrate preferences and/or in the structural stability to that of the mature form) or to other binding sites of interacting partners or natural modulators. Transiently occurring allosteric binding sites of the precursor represent another interesting alternative. It is not only the inhibition but also the up-regulation or premature protease activation that is detrimental for the virus. Viral proteases are often cytotoxic and thus their increased activity in infected cells could help to eliminate virus-bearing host cells. Such a possibility is interesting mainly for chronically infecting viruses. Regardless of the chosen approach, viral protease precursors offer a promising frontier for drug discovery.
